# ACDB: An Antibiotic Combination DataBase

**DOI:** 10.3389/fphar.2022.869983

**Published:** 2022-03-18

**Authors:** Ji Lv, Guixia Liu, Wenxuan Dong, Yuan Ju, Ying Sun

**Affiliations:** ^1^ College of Computer Science and Technology, Jilin University, Changchun, China; ^2^ Key Laboratory of Symbolic Computation and Knowledge Engineering of Ministry of Education, Jilin University, Changchun, China; ^3^ College of Computer Science, Sichuan University, Chengdu, China; ^4^ Sichuan University Library, Sichuan University, Chengdu, China; ^5^ Department of Respiratory Medicine, The First Hospital of Jilin University, Changchun, China

**Keywords:** antimicrobial resistance, antibiotic combinations, synergy effect, fractional inhibitory concentration index, database

## Introduction

Antimicrobial resistance (AMR) is a pressing public health concern (Lv et al., 2021; Murray et al., 2022). It is estimated that by 2050, 10 million people will die from antimicrobial resistance, leading to economic losses of 100 billion U.S. dollars annually (O’neill, 2014). Much of this problem comes from the lack of innovation in antibiotic discovery. Most antibiotics were discovered within a few decades after the Second World War (Lv et al., 2021). Moreover, many pharmaceutical companies have abandoned projects searching for new antibiotics due to costs and market regulation. Therefore, alternative strategies for the treatment of bacterial infections are urgently required.

Drug combinations provide an effective strategy to combat antimicrobial resistance (Ryall and Tan, 2015; Tyers and Wright, 2019; Lv et al., 2021). Compared to monotherapy, drug combinations can provide improved efficacy with lower doses and/or slow the development of resistance (Tyers and Wright, 2019) and thus attracts the attention of both researchers and pharmaceutical companies (Ramsay et al., 2018). Previously, the effectiveness of drug combinations was determined through clinical trials. However, this approach is both expensive and time-consuming. In recent years, with the development of high-throughput screening (HTS) technology (Bajorath, 2002), it has been possible to simultaneously evaluate hundreds of drug combinations. Therefore, datasets of drug combinations are becoming more prevalent, and they also present an excellent opportunity for data-driven artificial intelligence (AI) models. However, these existing models often have “curse of dimensionality” problems, resulting in moderate accuracy (Chandrasekaran et al., 2016; Mason et al., 2017). Specifically, if the amount of available training data is fixed, then overfitting occurs if the number of features is much greater than the number of training sets. Therefore, a database to collect and integrate the growing antibiotic combination data is required. Wu et al. (Wu et al., 2021) summarized existing databases for drug combinations. These databases focus on a specific field, such as, anticancer (Liu et al., 2019; Seo et al., 2020; Zheng et al., 2021) or anti-fungal drug combinations (Chen et al., 2014). To our knowledge, a database for antibiotic combinations is not yet available.

**FIGURE 1:**
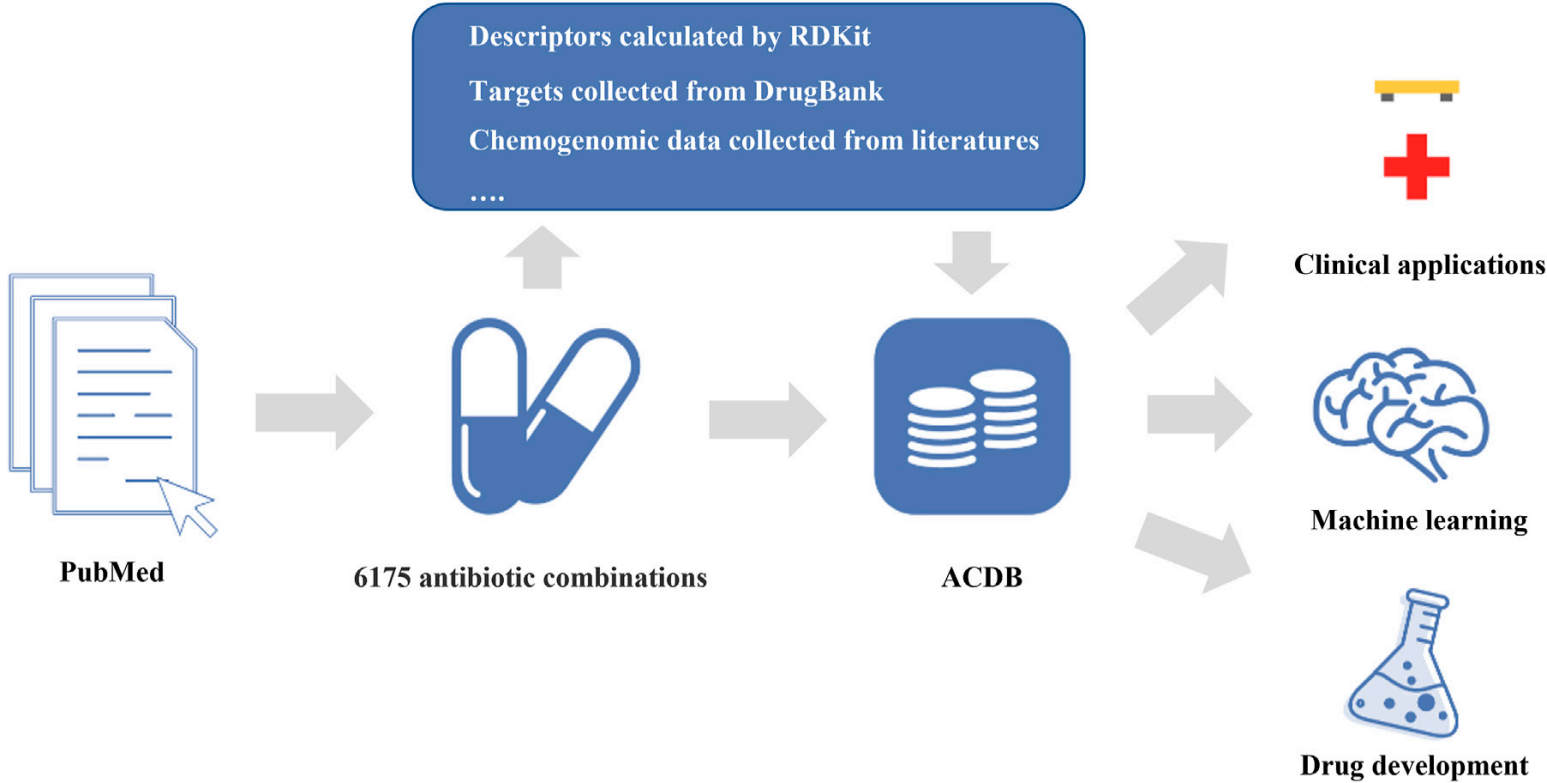
Database sources, build process and application.

In this study, we constructed a comprehensive database (Antibiotic Combination DataBase, ACDB) focused on antibiotic combinations (Figure 1). This current release of ACDB includes 6,175 antibiotic combinations that were manually collected from the literature, covering 304 unique compounds and 460 bacterial strains. In addition, we also provided descriptors (e.g., LogP, molecular weight), molecular fingerprints (e.g., MACCS Keys, Morgan fingerprints), targets for each compound and chemogenomic data. Such data are a valuable resource for data-driven AI models. We developed a user-friendly website where the data can easily be acquired and analyzed further by users. In this way, it should be useful in predicting new antibiotic combinations and, in turn, combatting antimicrobial resistance.

## Methodology

### Data Sources of Antibiotic Combinations

Many studies of antibiotic combinations have been reported in PubMed. To obtain high-quality antibiotic combinations, we manually collected literature-reported antibiotic combinations from thousands of studies. Specifically, the keywords “antibiotic (s),” “fractional inhibitory concentration,” “synerg*,” and “combination (s)” were used to retrieve related literature in PubMed, and we finally obtained 6,175 antibiotic combinations from 6,121 publications (Dec. 2021).

### Properties of Compounds

We then filtered out duplicate content and 304 unique compounds were obtained. We normalized them into a standard format (PubChem CID) because they are well-known and easily linked to external databases. Simplified Molecular Input Line Entry System (SMILES) of each compound was obtained from PubChem, and we used SMILES to calculate descriptors (*e.g.*, LogP, molecular weight, Lipinski’s five rules, Table 1). Moreover, we provided an additional Python script that converts SMILES into Protein Data Bank (PDB) format for download and further studies (molecular docking and/or molecular dynamics simulations). Targets of each compound were collected from DrugBank (Wishart et al., 2007).

**TABLE 1:** Overview of molecular descriptors.

Abbreviation	Description
LogP	The LogP of the drug (one of the Lipinski’s five rules)
HBA	The number of H-bond acceptors (one of the Lipinski’s five rules)
HBD	The number of H-bond donors (one of the Lipinski’s five rules)
TPSA	The drug polar surface area of drug
ROTB	The number of rotatable bonds (one of the Lipinski’s five rules)
AROM	The number of aromatic rings
ALERTS	The number of structural alerts

### Website and Server

ACDB is a relational database. It is hosted on a cloud server (CentOS Linux release 8.5.2111), which employs Apache (version 2.4.37) and MySQL (version 8.1) as the web and database server, respectively. The website is built with PHP, HTML and CSS and it can be freely accessed at http://www.acdb.plus.

## Organization of ACDB

There are six sections (Home, About, Download, Contact, Help and Visualization) on the ACDB website. A user-family retrieval system for antibiotic combinations was available on the homepage. The retrieval system allows users to find antibiotic combinations of interest to them. The About page contained the overview and motivation of ACDB. The Download page contained many useful datasets. These datasets can be used for AI-based models, and we will cover these usages in detail later. On the Help page, users can learn how to use ACDB. On the Visualization page, users can upload the dose-effect matrix, and then it will be fitted with the Loewe model. Finally, a heatmap and an isosurface will be shown on the webpage. If users have any further questions, they can find our contact information in the Contact page.

## Potential Applications of ACDB

ACDB aims to help researchers obtain datasets of antibiotic combinations for further applications and analysis. ACDB contains a large amount of data on structure, pharmacology, and well-documented antibiotic combinations, which contribute to the development of combination therapy. In this section, we list some potential applications of ACDB.

### AI-Based Prediction of Antibiotic Combinations

Since experimental approaches for distinguishing antibiotic combinations are expensive and time-consuming, an increasing number of researchers are using machine learning methods to predict potential antibiotic combinations (Chandrasekaran et al., 2016; Mason et al., 2017; Wu et al., 2021). To make it easier for users, ACDB incorporated a series of features including Lipinski’s five rules, molecular fingerprints (e.g., MACCS Keys, Morgan fingerprint) and chemogenomic data. These features have been successfully applied in previous work to predict potential antibiotic combinations. For example, Yilancioglu *et al.* (Yilancioglu et al., 2014) investigated the relationships between Lipinski’s five rules and synergistic drug combinations, and they found a significant correlation (*r* = 0.51, *p* = 3.6 × 10^–3^) between synergistic drug combinations and lipophilicity. Chandrasekaran et al. (Chandrasekaran et al., 2016) used chemogenomic data of *Escherichia coli* (*E. coli*) as features to build an AI-based model and subsequently predict new antibiotic combinations (AUC for synergy = 0.79). On the download page, users can obtain the entire chemogenomic data of *E. coli* in 324 conditions (Nichols et al., 2011). For other bacterial strains, orthologous genes can be obtained from OrtholugeDB (Whiteside et al., 2012). The disadvantage of the method is that chemogenomic data are expensive to obtain. Computational features add another alternative for AI-based models. Mason et al. (Mason et al., 2017) used MACCS keys to build an AI-based classifier and obtained acceptable results (AUROC = 0.74). On the download page, we offered three types of molecular fingerprints of each compound (MACCS keys, Morgan fingerprint and topological pharmacophore fingerprints) for users to choose.

### Investigate Mechanisms of Antibiotic Combinations Based on Network Analysis

While machine learning models can offer satisfactory outcomes, the mechanisms underlying the synergy effect are still poorly understood. As such, mechanism-driven methods are needed in order to predict antibiotic combinations. Network pharmacology provides a new paradigm to explore intricate relationships between drugs, genes, and diseases (Hopkins, 2008). ACDB provides targets for each compound and several common protein-protein interaction (PPI) networks. Furthermore, Cytoscape (Shannon et al., 2003) and the Python package networkx can be used to draw the PPI network and calculate topological parameters (*e.g.* degree, betweenness, eigenvector centrality) for each node. Zou et al. (Zou et al., 2012) used these topological parameters to explore the underlying mechanisms of drug combinations. Network-based proximity (Cheng et al., 2019; Lv et al., 2022) can also be used to measure the relationship of two drugs. Based on the ACDB, comprehensive studies of antibiotic combinations at the system level can be undertaken.

### References for Clinical Treatment

Pharmacologically, an antibiotic combination may produce synergy effect, additive effect, and antagonism effect (Cokol et al., 2011). Every antibiotic combination has its own advantages. For synergistic antibiotic combinations, they are frequently used in clinics because they can provide improved efficacy at lower dosages (Yeh et al., 2009). The combination of trimethoprim and sulfamethoxazole, for example, can interfere with folic acid synthesis in a synergistic way (Yeh et al., 2006). For antagonistic antibiotic combinations, they have been shown to slow down the evolution of AMR (Chait et al., 2007; Michel et al., 2008). However, the potency of antibiotic combinations is not immutable and it is affected by metabolic conditions (Cokol et al., 2018), bacterial strains (Chandrasekaran et al., 2016), *etc*. This is one of the important drivers for development of ACDB. Through the “Organism Search” in ACDB, users can obtain a series of species-specific antibiotic combinations and their efficacy. Undoubtedly, ACDB combined with antimicrobial susceptibility testing can help clinicians tailor treatments based on the pathogen microenvironment and the patient’s condition.

## Conclusion

We introduce a freely available database focusing on antibiotic combinations. To our knowledge, ACDB is currently the only database utilizing this approach. ACDB contains a great number of well-documented drug combinations and structural, physicochemical, pharmacological and chemogenomic data. It should benefit the performance of AI-based models and to explore the mechanism of synergy effects. In future versions, combinations of antibiotics, human-targeted drugs, and plant extracts and more applications will be incorporated into this web-based program.

## Data Availability

ACDB can be freely available at http://www.acdb.plus and we will update it annually.
